# Book Reviews

**Published:** 1903-09

**Authors:** 


Medical and Surgical Uses of Electricity. By A. D. Rockwell, A. M., M. D., formerly Professor of Electrotherapeutics in the New York Post-Graduate Medical School and Hospital. Illustrated, new edition. New York: E. B. Treat & Company. 1903. (Price: cloth, $5.00.)

The author of this treatise and his associate, the late Dr. Beard, were the first to write intelligently or scientifically upon the therapeutic uses of electricity; at least, so far as this country is concerned, they were pioneers. Their books were frequently revised and new editions were issued from time to time, keeping pace with the progress that was making such rapid strides on this topic. Some seven or eight years ago the present author reconstructed the work, having had the old stereotype plates destroyed. The illustrations were newly drawn and much of the treatise was rewritten. The present edition has been revised still further, carrying forward all knowledge on the subject to the immediate present. Especially have the latest discoveries received appropriate and detailed attention. These include the *x*-ray, the Finsen light, vibratory therapeutics, and the high frequency currents, which are described and discussed in the last six chapters, and which comprise entirely new material.

The division of the general subject into sections is admirable, and they comprise electrophysics, electrophysiology, electrotherapeutics, and electrosurgery. Every known use of electricity that possesses any value whatever is described under these several heads. The illustrations, as in most works on electricity, are very largely given over to the portrayal of instruments and machines. It would seem important to enlarge the scope of this part of the treatise to the end, that it should become equally as famous for its accurate clinical portraits and engravings as it is for its other valuable material. However, from any viewpoint, it is a book that no person interested in the medical uses of electricity can afford to ignore. Its possession is a necessity to every physician who employs electricity in the treatment of disease.

TRANSACTIONS OF THE AMERICAN ROENTGEN RAY SOCIETY. Third annual meeting held at Chicago, Ill., December 10 and 11, 1902. James B. Bullitt, M. D., Secretary. (Price: cloth, $1.25; paper, 75 cents.)

This society was organised for the purpose of putting on record the most striking successes of the new light science. The book contains 14 papers, all upon the subjects connected with radiotherapy. The use of the *x*-ray and its allies is becoming more and more common; so common, indeed, as to preclude the necessity of a national organisation on the part of those employing these agents. Used as it is in all departments of medical science it would not seem to demand any such distinction. The medical profession has already been nationally organised to suffocation. Let us not seek to add anything to the present alarming aggregate. G. W. W.

---

Twenty-ninth Annual Report of the Michigan State Board of Health. For the fiscal year ending June 30, 1901. Henry B. Baker, M. D., Secretary.

The reports of the Michigan State Board of Health have always been noted for accuracy and scientific value, this one being no exception to the rule. The section dealing with consumption is, perhaps, the most interesting in the book. It compares its prevalence with previous years from 1893, gives in distribution by counties in a schematic map of the state, and affords much further information, statistical and otherwise, relating to the disease and the methods adopted in dealing with it. The entire report reflects the greatest credit upon the secretary, under whose supervision it is prepared.

